# Engineering photosynthesis: progress and perspectives

**DOI:** 10.12688/f1000research.12181.1

**Published:** 2017-10-26

**Authors:** Douglas J. Orr, Auderlan M. Pereira, Paula da Fonseca Pereira, Ítalo A. Pereira-Lima, Agustin Zsögön, Wagner L. Araújo

**Affiliations:** 1 Lancaster Environment Centre, Lancaster University, Lancaster, LA1 4YQ, UK; 2 Max-Planck Partner Group at the Departamento de Biologia Vegetal, Universidade Federal de Viçosa, Viçosa, Minas Gerais, Brazil; 3 Departamento de Biologia Vegetal, Universidade Federal de Viçosa, Viçosa, Minas Gerais, Brazil

**Keywords:** photosynthesis, crop improvement, Rubisco, Calvin-Benson cycle, CCM, light-use efficiency

## Abstract

Photosynthesis is the basis of primary productivity on the planet. Crop breeding has sustained steady improvements in yield to keep pace with population growth increases. Yet these advances have not resulted from improving the photosynthetic process *per se* but rather of altering the way carbon is partitioned within the plant. Mounting evidence suggests that the rate at which crop yields can be boosted by traditional plant breeding approaches is wavering, and they may reach a “yield ceiling” in the foreseeable future. Further increases in yield will likely depend on the targeted manipulation of plant metabolism. Improving photosynthesis poses one such route, with simulations indicating it could have a significant transformative influence on enhancing crop productivity. Here, we summarize recent advances of alternative approaches for the manipulation and enhancement of photosynthesis and their possible application for crop improvement.

## Introduction

Photosynthesis consists of a series of biochemical reactions whereby plants use sunlight to reduce atmospheric CO _2_ into carbohydrates, releasing O _2_ as a byproduct. The first photosynthetic organisms appeared at least 2.5 billion years ago (Archean Eon) and were single-celled ocean-dwelling prokaryotes. Thus, photosynthesis was originally an aquatic-based process occurring in a strongly reducing atmosphere ^1^. The transition to a terrestrial environment and to an oxidizing atmosphere subsequently shaped the photosynthetic pathway into its current form ^2, 3^. Plant cells contain chloroplasts, which are organelles that originated from endosymbiosis of a cyanobacteria-like organism. Chloroplasts harbor the photosynthetic machinery and confer upon plants their characteristic green color. Sunlight within the visible spectrum is captured by chlorophyll and other accessory pigments and used to energize electrons derived from a water molecule in the thylakoid membrane of the chloroplasts. High-energy electrons are then transferred to carrier molecules, which can donate them for the reduction of gaseous CO _2_ to triose-phosphates in the chloroplast stroma. The enzyme responsible for the first step in CO _2_ fixation is ribulose-1,5-bisphosphate carboxylase/oxygenase (Rubisco). The Calvin-Benson cycle allows for the regeneration of the ribulose-1,5-bisphosphate molecule (RuBP) ^4^, whereas the fixed CO _2_ molecule moves on to anabolic pathways for sucrose and starch biosynthesis.

**Figure 1.**
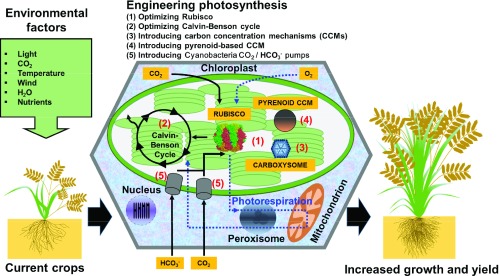
A number of routes are being studied to improve photosynthesis in crop plants. These include (1) improving the efficiency of the primary CO _2_-fixing enzyme Rubisco, (2) optimizing elements of the Calvin-Benson cycle, (3) introducing the carboxysome-based carbon concentrating mechanism (CCM) from cyanobacteria, (4) introducing an algal pyrenoid CCM, and (5) improving the photochemical response of photosynthesis to rapid changes in light conditions. Further studies are also related to attempts to convert C _3_ crops such as rice to the more efficient C _4_-type photosynthesis.

The high energetic value of sucrose and starch drove the domestication of plants to create crops, spawning the agricultural revolution and the transition from a hunter-gatherer to the current agricultural-industrial society ^5^. It is thus clear that photosynthesis is a cornerstone of human civilization and, as such, the object of intense basic and applied research in the face of mounting pressure to feed an increasing population ^6, 7^. Decades of research have provided a detailed picture of the intricacies of the photosynthetic process and suggested potential avenues for its improvement ^8^. A broad range of opportunities have been identified to improve photosynthetic efficiency; for recent detailed reviews, see 7, 9– 11. We now know, for instance, that owing to the complex interaction between physiological and environmental parameters photosynthetic rate does not directly extrapolate to whole plant growth rate ^12^. Breeders have managed to increase yields via processes that alter carbon partitioning rather than improving photosynthesis ^13^. Breeding better crops through improved photosynthesis is a long-sought goal but so far has remained unrealized because of the multiplicity of challenges involved ^12^. Here, we briefly review the current state of the ongoing efforts in molecular engineering to improve photosynthesis, plant growth, and yield ( Figure 1).

We start by summarizing recent efforts to optimize Rubisco performance. Rubisco is an ancient enzyme that evolved in a CO _2_-rich atmosphere devoid of O _2_. It is a slow (turnover rate of ~3–5 s ^-1^ compared to around >100 s ^-1^ for most enzymes) and error-prone enzyme (fixing O _2_ instead of CO _2_ in up to one-third of reactions) ^14^. The unavoidable side reaction with O _2_, oxygenation of RuBP, leads to the photorespiratory cycle, which “recycles” unproductive reaction products ^15^. However, this recycling comes at the cost of previously fixed carbon and a further loss of chemical energy ^16^. To make up for such shortcomings, plants generally contain large amounts of Rubisco (up to 50% of leaf total protein), which entails a large N-investment ^17^. Despite the significant natural variation in Rubisco catalysis and structure, it maintains a conserved, complex, catalytic mechanism that intrinsically imparts a trade-off between an enzyme specificity for CO _2_ over O _2_ and its catalytic speed. Next, we synthesize recent work aimed at optimizing other enzymes of the Calvin-Benson cycle. Theoretical and experimental data suggest that under non-Rubisco-limiting conditions other enzymes in the cycle begin to limit photosynthetic rate ^18^. We summarize recent progress on the manipulation of carbon concentrating mechanisms (CCMs), which are evolutionary solutions to counter Rubisco inefficiency. Well-known examples in plants include C _4_ photosynthesis and crassulacean acid metabolism (CAM) ^19, 20^. C _4_ and CAM photosynthesis are highly efficient processes ^21^; however, plants using these processes are relatively restricted in number within the plant kingdom ^22, 23^. Exciting recent developments in efforts to introduce CCMs from non-plants are also discussed and summarized in Figure 1. We additionally emphasize the challenges and opportunities to further understand the complex interplay between photosynthesis and related metabolic processes that has limited success in the manipulation and improvement of photosynthesis.

## Engineering Rubisco

The observed inefficiencies of Rubisco such as a slow CO _2_-fixation rate and poor specificity for CO _2_ over O _2_ have made it a key engineering target to improve photosynthesis in crop plants (for reviews, see 8, 17, 24). Whilst there is a good understanding of the reaction mechanism ^25, 26^, engineering efforts have yet to produce the holy grail of a “super Rubisco”, as efforts to modify one aspect of its catalytic biochemistry typically come at a cost to another ^8, 17^. A notable discovery was that a single amino acid mutation acted as a catalytic “switch” to convert Rubisco from different *Flaveria* species from a “C _3_ style” enzyme to a “C _4_ style” and vice versa ^27^. This clearly demonstrates the potential for manipulating the performance of Rubisco with targeted changes inferred from comparisons of natural diversity in enzyme sequence and catalysis. This has spurred a recent influx of data on the natural diversity of Rubisco in a bid to identify amino acid changes that can improve its catalysis in plants ^28– 36^. Insights into how Rubisco and other photosynthetic traits have co-evolved are critical to guiding kinetic characteristics for an improved crop Rubisco under differing environmental scenarios (e.g. 37, 38). Rubisco screens extending outside of the plant realm have also proved highly informative. For example, the Rubisco from diatom and haptophyte microalgae has undergone differing selection pressures that see its kinetic properties diverge from the canonical trade-off between catalytic rate and affinity for CO _2_^39, 40^, providing possible new areas of exploration for improving Rubisco efficiency ^41^.

A key challenge in engineering Rubisco in plants is that it is a hefty 550 kDa hexadecameric complex comprising eight large subunits ( *ca*. 52 kDa each) and eight small subunits ( *ca*. 13 to 15 kDa). It is produced through an exquisite synthesis and assembly process that is dependent on a number of nucleus- and chloroplast-encoded components ^42^. Typically, research has focused on the chloroplast-encoded large subunits, which contain the catalytic sites and can be routinely manipulated in tobacco for functional genomic studies ^24, 43^. More recent reports have highlighted the impact on catalysis of manipulating the small subunits ^44– 47^. The nuclear-encoded small subunit gene family (RbcS) is therefore a growing target for engineering, as nuclear transformation is already established in many species compared to the relatively few species amenable to chloroplast transformation ^42, 48^. An alternative approach to altering the extant Rubisco in a crop species is to replace it with better-performing natural variants, although this is subject to similar technical limitations. Introduction of foreign Rubisco is typically hampered by the complicated assembly requirements of the enzyme in the chloroplast (see 49), though important advances have been made in co-engineering introduced Rubisco alongside assembly chaperones ^50^. Besides efforts to introduce a cyanobacterial CCM into higher plants (see below), the Rubisco from *Synechococcus elongatus* has now been successfully introduced into tobacco ^51^ and can support growth at elevated CO _2_^52^. Whilst supporting much higher catalytic rates than higher plant Rubisco, the Rubisco from cyanobacteria has lower CO _2_ affinity and specificity for CO _2_ over O _2_. Thus, for cyanobacteria Rubisco to support plant growth at ambient CO _2_ levels, co-engineering of a functional CCM is required ^53^.

## Calvin-Benson cycle optimization

It has long been recognized that other enzymes in the Calvin-Benson cycle represent viable targets to accelerate carbon fixation in plants (reviewed in 9, 18, 54– 56). Recently, efforts have built on prior success in overexpressing SBPase (sedoheptulose-1,7-bisphosphatase) to improve growth in tobacco and rice ^55, 57, 58^. These efforts have now expanded to include other Calvin-Benson enzymes in a combinatorial manner to avoid creating new bottlenecks in different parts of the cycle ^59, 60^. Increases in plant biomass have also been obtained by jointly manipulating genes in the Calvin-Benson cycle and the photorespiratory pathway ^60^. An alternative approach has been to avert the CO _2_ and energy costs of photorespiration by introducing synthetic “photorespiratory bypass” pathways in the chloroplast that direct CO _2_ release in the proximity of Rubisco ^61– 64^. The potential benefits and caveats of the differing bypass strategies are reviewed in 65, 66.

In addition to the overexpression of native enzymes ( Figure 1), a number of studies have also shown effects through the expression of foreign membrane transporter proteins in both model ^59, 67– 69^ and crop species such as soybean and rice ^70, 71^. These genes derived from cyanobacteria include the frequently studied but functionally-unknown membrane transporter protein IctB ^72^. Introducing IctB has not always improved crop growth: for example, in one case changes were noted in photosynthetic rate without an increase in biomass ^71^. In contrast, IctB expression improved growth in crop species such as wheat in the glasshouse ^73^ and soybean in the field ^70^. Importantly, extended field testing under future environmental scenarios through free-air CO _2_ enrichment (FACE) experiments enables validation of *in silico* and glasshouse-based predictions. For example, in soybean one such manipulation has been shown to counteract some of the negative impact of future climate on yield ^74^. These inconsistent findings underscore the frequent discontinuity in crop yield predictions between glasshouse and field trials and the importance of FACE field studies for screening the suitability of natural and engineered crops for future climates.

## Optimizing response to changes in light-use efficiency

Major losses of energy conversion during plant biomass formation occur during light absorption and the photochemical reactions ^75^. Dramatic increases in tobacco growth rate in the field were obtained recently by improving the rate of relaxation of photoprotection (non-photochemical quenching, NPQ). NPQ is the process by which plants dissipate excess light as heat when they receive more than they are capable of using ^76^. Modeling suggested this as an area where there is room for improvement ^77, 78^. Combinations of genes involved in photoprotection were transformed into tobacco and those that accelerated the relaxation of NPQ increased the rate of biomass production by as much as 20% in glasshouse trials and around 15% in duplicated field trials ^76^. The modifications involved introducing multiple genes expressing key components of the xanthophyll cycle and the PsbS subunit of photosystem-II. This combination allowed the plants to more quickly adapt to fluctuating light, turning off photoprotection and using light for photosynthesis rather than continuing to dissipate it as heat after a decrease in light level. The conservation of NPQ across plants suggests this approach may also serve to improve the growth of other crops. However, the mechanisms allowing plant cells to cope with the excess energy, such as NPQ, tend to decrease the overall efficiency of energy storage, since surface cells exposed to the light dissipate much of the available energy whereas cells in the lower layers remain light limited ^79^. Thus, the temptation to increase the capacity to process the influx of energy by the photosynthetic apparatus is a challenge that must be pursued to ameliorate energy losses during plant photosynthesis (reviewed in 79). Increasing light energy capture might also be feasible by incorporating the bacteriochlorophylls found in many anoxygenic photosynthetic organisms into one of the photosystems in plants with the aim of extending the light absorption spectrum by plants to the far red region out to ~1,100 nm ^79^.

Changes in the antenna size of the photosystems have also been suggested as a route that could enhance solar conversion efficiency while reducing NPQ. This assumption is based on the fact that the antennae trap more light than can be used for photochemistry. Based on this, the theory is that in plants with a reduced number of light-harvesting pigments (e.g. chlorophyll and carotenoids) per photosystem, solar energy conversion efficiency could be significantly enhanced ^11, 80– 82^. Changes in the leaf optical properties would alleviate over-absorption and mitigate efficiency losses associated with wasteful dissipation of sunlight by the upper canopy. Furthermore, they could bring about enhancements of photosynthetic productivity due to greater transmittance of light to lower layers, thus improving canopy light distribution and canopy photosynthesis. This truncated light-harvesting antenna (TLA) concept ^80, 81^ has been successfully applied in microalgae ^83– 85^ and cyanobacteria ^86^. Recent work has shown that TLA enhances both photosynthesis and plant canopy biomass accumulation under high-density cultivation conditions in both tobacco ^82^ and rice ^87^. This approach presents intrinsic practical limitations and challenges ^81^, but ongoing research suggests that expanded efforts could ultimately optimize this exciting biotechnological process.

## Synthetic biology for CO _2_ fixation in non-plants

Synthetic photosynthesis has made exciting progress recently via the incorporation of non-native Calvin-Benson-like carbon assimilation in *Escherichia coli*. The highlight was the production of a bacterium that is capable of making sugars and other life-preserving metabolites from atmospheric CO _2_^88, 89^. Although at this stage both energy and reducing power are required through the oxidation of an external organic acid in an isolated metabolic module and thus no net carbon gain is achieved, this discovery clearly proves the potential of synthetic biology to optimize pathways of biotechnological significance and may even lead to new avenues for optimizing CO _2_ fixation in plants ^89^. Along such lines is the successful development of a synthetic carbon fixation pathway that functions efficiently *in vitro* but faces significant challenges for it to be compatible in a biological context ^90^.

## Introducing the C _4_ cycle in C _3_ crops

C _4_ photosynthesis has evolved independently of C _3_ photosynthesis in several angiosperm families during the last 25 million years in at least 66 independent events ^91^. Despite the frequency of these events, the evolution of C _4_ photosynthesis is not distributed evenly in the plant kingdom ^92^. This multiple parallel evolution appears to have occurred as an adaptive response to low atmospheric CO _2_ concentrations and high temperature ^22^. The transition from C _3_ to C _4_ plants requires the evolution of both morphological and physiological traits. Among these, the differentiation of photosynthetically active vascular bundle sheath cells, modification in the biochemistry of several enzymes, and increased intercellular and intracellular transport of metabolites are of pivotal significance. This makes the evolution of such a complex trait system in one single step highly unlikely ^93^. The first evidence of evolutionary intermediate C _3_–C _4_ forms was reported in the 1970s ^94^, and this spurred intensive efforts to understand the mechanistic bases of the transition from C _3_ to C _4_.

A better understanding of the initial events that occurred during the C _3_ evolution to C _3_–C _4_ intermediates and then to C _4_ plants can contribute to increasing photosynthetic efficiency in C _3_ plants. Whilst C _4_ photosynthesis requires considerable additional ATP, the plants benefit from enhanced biomass production and improvements in nitrogen and water use efficiencies. Using complementary approaches, including genome and transcriptome analyses, the international C _4_ rice consortium is working toward introducing the C _4_ mechanism into rice ^95^. This research initiative has already produced exciting results, including the identification of metabolite transporters and transcription factors ^95, 96^. Although the genes identified are potentially useful for engineering C _4_ rice, clearly further investigation is required. Additional examination of temporal, spatial, and environmental dynamics spanning C _3_ through C _3_–C _4_ intermediacy and true C _4_ species will no doubt be highly informative for identifying useful genes and regulatory components (e.g. 97, 98).

Recent efforts have expanded the number of genera studied beyond *Flaveria* species to include *Cleome* and *Moricandia*, two close relatives of the model C _3_ *Arabidopsis*, which both contain intermediate as well as true C _4_ species ^92, 99^. This provides a powerful opportunity to accelerate advances through comparison with the large amount of data already available for *Arabidopsis* in order to find the minimal genetic basis of C _4_ photosynthesis. This strategy has the growing potential to promote substantial increments in the yield of C _3_ crops usually cultivated in dry and hot areas ^95^. Importantly, at least some of the technical difficulties associated with separately isolating pure bundle sheath and mesophyll cells from C _3_ plants have been overcome in *Arabidopsis* ^100^. A very large-scale analysis of the bundle sheath translatome in *Arabidopsis* demonstrated its high similarity with the translatome in the root pericycle cells ^100^. This study not only provides the foundation to enhance our understanding of the evolutionary function of bundle sheath cells in C _3_ plants but also indicates that a highly similar and conserved regulatory network might sustain bundle sheath and pericycle cell functionality in *Arabidopsis thaliana.* Although several open questions remain, it seems clear that these types of studies are likely to be key to understanding the genetic triggers needed to re-organize the anatomy, gene expression, and biochemistry within C _4_ plants, possibly paving the way toward producing C _4_ rice.

Whilst perhaps less advanced than the efforts to understand and engineer C _4_ photosynthesis, a number of groups are working to better understand the key elements required for CAM photosynthesis ^101, 102^. CAM plants are typically highly efficient in their use of water, and engineering of CAM into food or bioenergy crops may prove most beneficial by improving crop water use efficiency and expanding the land area capable of supporting agriculture ^103^. CAM species may also serve as a suitable source of high-temperature-adapted enzymes of potential application in photosynthetic engineering ^104^.

## Introducing non-plant CCMs into C _3_ plants

One approach to improving plant photosynthesis is using engineering to implement CCMs from other photosynthetic organisms such as cyanobacteria or algae. While components of the CCM in cyanobacteria and algae can differ, both systems function to create a high-CO _2_ environment around Rubisco to overcome its catalytic shortcomings. Among the core components of CCMs are inorganic carbon transporters and the co-localization of carbonic anhydrase (catalyzing the interconversion of HCO _3_^–^ and CO _2_) around Rubisco to maintain high CO _2_ levels. In some instances, this association is contained within a protein micro-compartment to limit CO _2_ escape and ensure high HCO _3_^–^ levels can be sustained in the cytosol.

## Cyanobacterial CCM

Amongst the CCMs currently being engineered into plants, the cyanobacterial carboxysome-based system has made the most striking progress in recent years. With a stronger—but still incomplete—understanding of the construction of this bacterial microcompartment (for in-depth reviews, see 72, 105– 109), significant progress has been made in assembling partial carboxysomes in higher plants using tobacco as a model system ^110^. Lin and colleagues ^111^ demonstrated the assembly of various complex structures by expressing as few as three β-carboxysome proteins. As mentioned above, parallel work on introducing a cyanobacterial Rubisco into tobacco was also successful ^51, 52^, with plants expressing cyanobacteria Rubisco and either the chaperone RbcX or the carboxysomal CcmM35 protein viable at elevated CO _2_. These are key advances to build upon through the addition of further components to assemble a fully functional carboxysome shell ^53^, and combining these to localize Rubisco inside the carboxysome will be a critical next step toward functionality. The internal components such as CcmM35 are thought to be important for the true icosahedral structure to be formed ^111, 112^. The ability to assemble bacterial microcompartments also has implications beyond crop productivity ^113^.

An important consideration for introducing a full cyanobacterial CCM into higher plants is creating a compatible HCO _3_^–^/CO _2_ environment by concentrating CO _2_ inside the carboxysome shell, removing stromal carbonic anhydrase, and introducing HCO _3_^–^ pumps to increase the bicarbonate concentration in the stroma ^53, 112, 114^. Progress has been made in attempting to introduce transporters into tobacco ^67, 115^, and issues related to correct localization are being targeted through a better understanding of transit peptides (e.g. 115, 116). Recent progress in other bacterial microcompartments beyond the cyanobacterial carboxysome are also providing key insights for targeting protein localization and the assembly of these complex structures (reviewed in 110).

## Pyrenoids

Although engineering an algal pyrenoid-based CCM into higher plants is less developed than cyanobacteria CCMs ^112^, there have recently been a number of key discoveries related to pyrenoid structure and function in the model *Chlamydomonas reinhardtii* (see 105, 109, 115, 116). Discoveries such as the highly disordered linker protein essential pyrenoid component 1 (EPYC1) that pulls Rubisco together ^108^ and that a loop structure on the Rubisco small subunit is necessary for pyrenoid formation ^117^ supply targets for engineering in plants. For example, mutagenesis of this loop region has shown its modification has no effects on Rubisco catalysis in *Arabidopsis* ^45^. Recent advances in the availability of a mutant Chlamydomonas library for functional studies ^118^ will likely accelerate advances in understanding, and engineering, algal pyrenoids. Increasing data on the CCM of other non-green micro-algae may also help better understand pyrenoid function and structural diversity ^40, 119^.

## Future perspectives and directions

Conventional crop breeding has thus far been sufficient to avert the dire Malthusian predictions of food shortage for a growing human population. Most projections suggest that novel yield-enhancing solutions are needed to avoid global crop production reaching a plateau. Genetic manipulation has been used to successfully engineer simple traits, such as insect and weed resistance. More refined molecular tinkering holds the promise of spectacular gains if fundamental pathways are targeted. Photosynthesis is one such pathway.

Synthetic biology is making large steps in engineering alternate CCM mechanisms into higher plants, in addition to efforts in manipulating elements of the Calvin-Benson cycle already present. Although mesophyll conductance to CO _2_ had been a relatively overlooked limiting factor until recently, it is now considered a promising potential target for increasing photosynthesis ^120^. It seems clear that expanded research efforts are currently required to build upon many of the technologies described above by, for example, enhancing our understanding of CAM metabolism and introducing alternative non-plant CCMs into C _3_ plants. Overcoming these challenges will require sustained investments in long-term research programs, with some of these research areas currently being advanced through large-scale, privately funded projects.

Ultimately, in the case of C _4_ rice, necessity for development in a C _4_ species as well as sufficiency for engineering C _4_ rice will need to be considered when determining gene function. Existing candidate regulators are currently being functionally validated, and this is ongoing: knockdown experiments in maize and setaria are examining necessity, while overexpression in rice is being used to scrutinize sufficiency. Future advances in engineering C _4_ rice will need to involve integrated analysis of these experiments together with further comprehension of the related gene regulatory networks. We posit that the successful integration of these different characteristics, as discussed above, coupled with the identification of the key regulators of C _4_ morphoanatomical pattern and the development of a strategy of how the C _3_ plant could be genetically altered allowing both the introduction and the establishment of the C _4_ pathway should be a significant breakthrough in the field of synthetic biology. Recent advances and ongoing incremental findings suggest that improved crop photosynthesis could assist towards feeding a growing population in the near future.
